# Editorial: Client Centric View of Population Health in the Digital Age—Making Healthcare Personal

**DOI:** 10.3389/fpubh.2022.941423

**Published:** 2022-06-22

**Authors:** Mohan Tanniru, Anupam Sule, Asha Shajahan

**Affiliations:** ^1^Mel and Enid Zuckerman College of Public Health, University of Arizona, Tucson, AZ, United States; ^2^St Joseph Mercy Oakland Hospital, Pontiac, MI, United States; ^3^William Beaumont School of Medicine, Oakland University, Auburn Hills, MI, United States

**Keywords:** population health, value cycle, client centric, community strategy, digital health

Population health has been viewed through various lenses (1), ranging from improving health outcomes for select patient groups to shaping the health of an entire population. Policies and practices designed to shape the health and wellbeing of client populations cannot ignore the influence of their ecosystem, often determined by social, cultural, economic, biological, and environmental factors (2). This calls for tailoring preventive as well as care transition practices to individual clients within their distinct ecosystem to improve health outcomes (3). There is a call for public health transformation to move from the epidemiological and policy design and development era to Public Health 3.0, where the focus is on engaging cross-sector collaboration and system level action to deliver tailored care to client populations as a part of community strategy (4). With the growing use by client populations of personalization technologies in the digital age, it is imperative that public health transformation extend their digital platform. Clients can self-manage their health and partner with multiple clinical and non-clinical actors in the community. The World Health Organization emphasizes the need for using technological tools and enabling platforms to extend care into client population ecosystems (5).

Such a client centric view of population health in the digital age calls for a service lens that integrates the activities of clients, health systems (hospitals and public health agencies) and other inter-organizational partners. The service lens shown in Figure 1 has three value cycle components: value creation, value fulfillment, and value in use assessment (6). This is used to ensure that community strategies continue to evolve with the changing ecosystem of all stakeholders involved. Client ecosystems are influenced by culture and by the changing vulnerabilities of client populations (7–9), which influence clients' perception of value created and value in use. This calls on all community actors (health systems and inter-organizational partners) to play a role in understanding and co-creating value with clients (1) and use feedback from client's perceived value in use (3) to reevaluate and refine the community strategy quickly to improve health outcomes. Similarly, the changing ecosystem of the community actors influences community strategy to fulfill value (3), and this requires the continual alignment of their goals and performance drivers to ensure effective care delivery.

**Figure 1:**
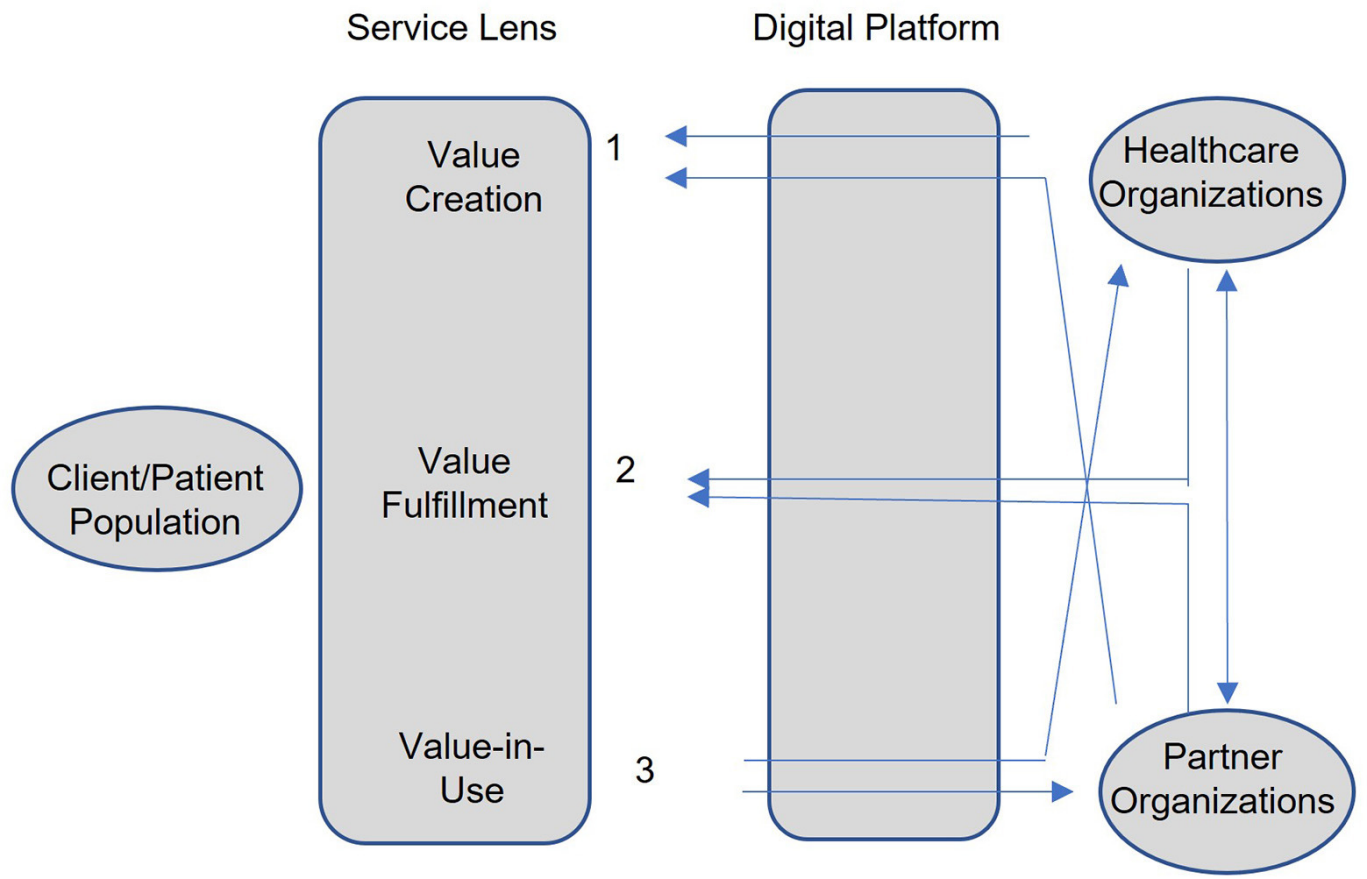
Community strategies using value lens and digital platform.

This special issue brings together five papers that highlight both the challenges and opportunities in transforming population health using community strategies that focus on various aspects of value cycle activities by leveraging technologies. The first article looks to understand client behavior in their use of the Internet so that value creation can work to influence their behavior. The second article looks to assess the value in use perceived by clients when care is brought to their home. The third article looks to use effective information sharing within health systems to support value fulfillment to make care delivery address individual client needs. The fourth article calls for broadening the scope of value cycle activities to improve quality of life needs of client populations. The last article argues for new funding models to reward community actors to outcomes as opposed to processes used in value cycle activities.

The article, “Influencing Factors on College Students' Willingness to Spread Internet Public Opinion: Analysis Based on COVID-19 Data in China,” tries to learn about the social and emotional factors that contribute to client (student) participation in the spread of information that can help formulate public opinion. Given the hesitancy among the public to follow vaccine protocols, learning about factors influencing client engagement in sharing information can help health systems co-create messaging to promote preventive practices.

The article, “Post-implementation review of the Himalaya Home Care project for home isolated COVID-19 patients in Nepal,” discusses how health systems made health care delivery personal by taking care delivery to client homes using intermediate actors (physician and nursing staff). While this reduces hospital wait times and addresses client barriers to care, the perceived value in use by clients must address community strategy goals. The article uses survey methodology to assess client feedback.

The article, “Precision Public Health for Non-communicable Diseases: An Emerging Strategic Roadmap and Multinational Use Cases,” discusses two case studies that illustrate how advanced technologies and digital platforms are used by hospitals and public health agencies to share data at a macro and micro or population group level to precisely identify the health and social conditions of client population to assess risks and target care delivery practices. They suggest a roadmap to tailor community strategies to improve healthy patient behaviors.

The article, “Improving the effectiveness of healthcare: diagnosis-centered care vs. person-centered health promotion, a long forgotten new model,” argues for moving health systems to a person or client centric model to address population health. Given the role of social, cultural, and economic barriers influencing multiple needs, such as access to food, jobs, transportation, and environment all influencing health, the authors call for an integrated approach to address care delivery not only during emergencies but also to address chronic care management of aging populations.

Lastly, the article, “Digital Health Approaches for Improved Population Health Outcomes: Time for a Disease Vulnerability Matrix for Individuals and Communities?” argues for developing health interventions to address chronic care management on a continuum—from the early diagnosis of unhealthy behaviors that contribute to chronic disease to managing the disease itself. Such an approach calls for strategies and funding models that reward not just processes but outcomes proposed by the interventions.

In summary, the goal of this special issue is to explore the role of advanced technologies in tailoring healthcare to be client focused and personal. Such a client centric lens requires the sustained engagement of clients, so that their changing health or social conditions are continually monitored to tailor care delivery models outside a hospital with the use of multiple external partners. The service lens is used to frame the focus of client engagement in creating, fulfilling, and assessing value-in-use using an evolving digital landscape.

## Author Contributions

All authors listed have made a substantial, direct, and intellectual contribution to the work and approved it for publication.

## Conflict of Interest

The authors declare that the research was conducted in the absence of any commercial or financial relationships that could be construed as a potential conflict of interest.

## Publisher's Note

All claims expressed in this article are solely those of the authors and do not necessarily represent those of their affiliated organizations, or those of the publisher, the editors and the reviewers. Any product that may be evaluated in this article, or claim that may be made by its manufacturer, is not guaranteed or endorsed by the publisher.
